# Cell Fusion-Related Proteins and Signaling Pathways, and Their Roles in the Development and Progression of Cancer

**DOI:** 10.3389/fcell.2021.809668

**Published:** 2022-02-01

**Authors:** Hao Zhang, Hong Ma, Xiaohui Yang, Linlin Fan, Shifeng Tian, Rui Niu, Man Yan, Minying Zheng, Shiwu Zhang

**Affiliations:** ^1^ Graduate School, Tianjin University of Traditional Chinese Medicine, Tianjin, China; ^2^ Tianjin Union Medical Center, Nankai University, Tianjin, China; ^3^ Nankai University School of Medicine, Nankai University, Tianjin, China; ^4^ Graduate School, Tianjin Medical University, Tianjin, China

**Keywords:** cell fusion, cancer stem cells, polyploid giant cancer cells, syncytin, glial cell missing 1

## Abstract

Cell fusion is involved in many physiological and pathological processes, including gamete binding, and cancer development. The basic processes of cell fusion include membrane fusion, cytoplasmic mixing, and nuclear fusion. Cell fusion is regulated by different proteins and signaling pathways. Syncytin-1, syncytin-2, glial cell missing 1, galectin-1 and other proteins (annexins, myomaker, myomerger etc.) involved in cell fusion *via* the cyclic adenosine-dependent protein kinase A, mitogen-activated protein kinase, wingless/integrase-1, and c-Jun N-terminal kinase signaling pathways. In the progression of malignant tumors, cell fusion is essential during the organ-specific metastasis, epithelial-mesenchymal transformation, the formation of cancer stem cells (CSCs), cancer angiogenesis and cancer immunity. In addition, diploid cells can be induced to form polyploid giant cancer cells (PGCCs) *via* cell fusion under many kinds of stimuli, including cobalt chloride, chemotherapy, radiotherapy, and traditional Chinese medicine. PGCCs have CSC-like properties, and the daughter cells derived from PGCCs have a mesenchymal phenotype and exhibit strong migration, invasion, and proliferation abilities. Therefore, exploring the molecular mechanisms of cell fusion can enable us better understand the development of malignant tumors. In this review, the basic process of cell fusion and its significance in cancer is discussed.

## Introduction

Cell fusion is involved both in physiological and pathological processes. According to the cell type, cell fusion can be categorized as homogenous or heterogeneous. Furthermore, cell fusion can be divided into total fusion and hemifusion according to whether the mixing of cell contents occurs. The basic processes of cell fusion include cell membrane fusion, cytoplasmic mixing, and nuclear fusion (Li et al., 2021). In addition, there are other forms of fusion that involve intercellular structures, such as entosis, which is a cell-in-cell structure where one cell is ingested by another, that can either play a pro-tumorigenic or tumor suppressor role (Krishna and Overholtzer, 2016). Fusion was shown to occur between two or more cells after membrane merging and cytoplasmic mixing, forming heterokaryons (multinuclear cells) or synkaryons (mononuclear cells) (Shabo et al., 2020). The mononuclear daughter cells of the two resulting hybrid cells were found to express all the chromosomes of the parental cells. Synkaryotes are formed by mixing and redistributing parental chromosomes to daughter cells through cell division, nuclear membrane separation, and recombination.

Following fusion, the hybrid cell obtains a new phenotype and becomes polyploid, in which the genome has more than two sets of chromosomes. Polyploid cells can be found in certain physiological or pathological stages, such as growth, development, aging, stress, cancer, and other diseases. The fusion of heteromorphic cells plays an important role in tissue development and disease pathogenesis (Ogle et al., 2005). In this review, cell fusion-related proteins, signaling pathway, and the role of cell fusion is elaborated. In cancer, cell fusion is associated with the progression of malignant tumors including organ-specific metastasis, epithelial-mesenchymal transformation (EMT), the formation of cancer stem cells (CSCs), cancer angiogenesis and cancer immunity.

## The Process of Cell Fusion

Human cells are coated with phospholipid bilayers separated from the extracellular environment. The occurrence of cell fusion requires morphological reconstruction of the cell membrane and merging of the cell contents. When under certain conditions, fused cells release proteins that transmit and receive signals, thus enabling them to judge the surrounding environment and the fusion target. Fusogens are a class of proteins that have been identified as necessary and sufficient for mediating cell fusion through diverse mechanisms. Some fusions are controlled by a single fusogen, while other fusions depend on several proteins that either work together throughout the fusion process or work in tandem to complete the process (Brukman et al., 2019). During myoblast fusion, Myomaker is involved in membrane hemifusion and Myomerger plays an important roles for fusion pore formation (Leikina et al., 2018). The functional independence reveals that myomaker and myomerger could serve as single fusogens. Hapless 2/generative cell-specific protein 1(HAP2/GCS1) functions in late stages of gamete fusion (Liu et al., 2008). In *C. elegans*, EFF-1 and AFF-1 independently mediate auto-fusion of cells (Rasmussen et al., 2008). However, various membrane-active factors and relevant mechanisms currently remain unidentified (Petrany and Millay, 2019).

**FIGURE 1:**
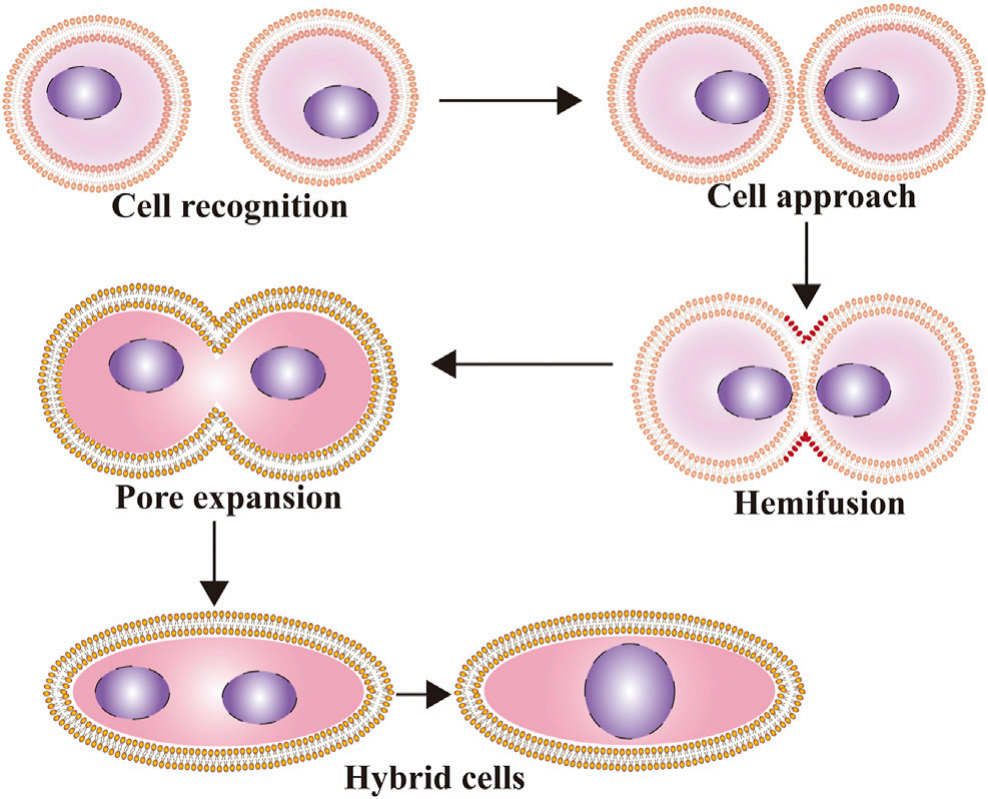
The process of cell fusion. The whole fusion process can be divided into three steps: preparation for fusion, membrane approach under the action of fusogens, and constitution of new cells with lipidic rearrangements.

The whole fusion process can be divided into three steps: preparation for fusion, membrane approach under the action of fusogens, and constitution of new cells with lipidic rearrangements (Hernández and Podbilewicz, 2017). The first step of cell-cell fusion depends on reception and response to extracellular signals for cell differentiation, followed by cell-cell recognition and interaction. Then, cells adhere together tightly as the distance between them decreases to less than 10 nm, while fusogens act in the final approach between membranes. In the second step, under the mediation of cell fusogens, the fusion of cell membranes undergoes three morphological changes: dehydration (Vargas et al., 2014), hemifusion (including unilateral and bilateral fusion) (Lee et al., 2015), and pore opening and expansion. In the last step, the cell membranes are integrated into a new single ring sharing all cytoplasmic and genetic material (Hernández and Podbilewicz, 2017) (Figure 1). However, in some cases, the process is aborted before pore opening and expansion occur, and the cell remains in the hemifusion phase so that its cellular content cannot be contacted and shared (Zawada et al., 2018). Symeonides et al. reported that HIV-1-induced cell-cell fusion could be blocked by tetraspanins at the transition stage from hemifusion to pore opening, leading to fusion failure (Symeonides et al., 2014).

### Cell Fusion-Related Proteins and Signaling Pathways

An increasing number of studies have shown that pro-fusion proteins play an important role in the cell fusion process. Cellular fusogenic proteins and actin-propelled membrane protrusions are necessary for the initiation of cell-cell fusion. Syncytin-1 (acquired by humans 19–28 million years ago) (Cáceres and Thomas, 2006) and syncytin-2 (acquired by humans 40 million years ago) (Blaise et al., 2005) are two important proteins that may promote cell fusion through the α-helix fusion mechanism. The α-helical fusion mechanism is found in target-soluble N-ethylmaleimide-sensitive factor attachment protein receptor (T-SNARE) and vesicular-SNARE (V-SNARE) (Larsson et al., 2008), which mediate intracellular membrane fusion through the formation of α-helical bundles, leading to attachment and membrane merging (Wickner and Schekman, 2008). Engineered flipping of T-SNARE and V-SNARE into the cell membrane surface can promote cell fusion (Chen and Olson, 2005; Larsson et al., 2008; Wu et al., 2017). Syncytin 1, syncytin 2, and their receptors are highly expressed in different cancers, suggesting that cell fusion may play an important role in the occurrence and development of cancer (Maliniemi et al., 2013; Huang et al., 2014; Yu et al., 2014; Fu et al., 2021).

#### Syncytin-1 and Cell Fusion

Syncytin-1 is a membrane glycoprotein encoded by the ENV gene of the human endogenous retrovirus (HERV) W family. It was the first fusion-promoting protein found to be involved in syncytiotrophoblast cell formation. Syncytin-1, which is composed of a surface subunit (SU) and transmembrane subunit (TM), is usually expressed in the placenta under certain physiological conditions. After the SU of syncytin-1 combines with the type D retrovirus receptor, which is a sodium-dependent type 2 neutral amino acid transporter (ASCT-2), the phospholipid bilayer structures of the two trophoblast cells become closely bound by the transmembrane subunit’s conformational change (Cheynet et al., 2005; Podbilewicz, 2014; Li and Karlsson, 2016). The TM also has a highly conserved domain, called the immunosuppressive domain (ISD), which can induce severe immunosuppression of host cells and induce cancers (Mangeney et al., 2007). The main function of syncytin-1 is to promote the fusion of mononuclear trophoblast cells into multinuclear syncytiotrophoblast cells and participate in the immunological regulation of the mother-fetal interface, which is an important condition for embryonic development. Syncytin-1 is also involved in other important cell fusion processes in humans, such as myoblast fusion (Bjerregard et al., 2014) and osteoclast formation (Søe et al., 2011). Bone resorption osteoclasts are equally large multinucleated cells, which are formed by the fusion of mononuclear precursors with mononuclear cells (Loutit and Nisbet, 1982).

Abnormal expression of syncytin is closely related to CSCs, as well as the development and progression of various tumors (Medvinsky and Smith, 2003; Larsson et al., 2007; María et al., 2016; Matteucci et al., 2018; Liu et al., 2019; Li et al., 2021). Antisense oligonucleotide-induced downregulation of syncytin-1 in breast cancer cells and endothelial cells expressing the syncytin-1 receptor, ASCT-2, partially inhibited spontaneous cell fusion *in vitro*. However, neither antisense oligonucleotide treatment nor a syncytin blocking peptide could completely inhibit the fusion of cancer cells with endothelial cells, suggesting that syncytin-1 is not a unique fusogenic protein expressed by cancer cells (Bjerregaard et al., 2006).

#### Syncytin-2 and Cell Fusion

Syncytin-2 is also an endogenous retrovirus gene product and an important fusion protein that contributes to the formation of placental syncytiotrophoblast cells (Tug et al., 2020). Like human syncytin-1, human syncytin-2 requires a 3′ untranslated region (3′-UTR) for efficient gene expression and retains a post-transcriptional regulatory element (SPRE). Insertion of SPRE significantly increased the expression of the reporter gene [Human immunodeficiency virus type 1 group-specific antigen (Gag)] without affecting the number of nuclear or cytoplasmic transcripts (Kitao et al., 2019). Syncytin-2 is a newly discovered placental membrane protein with induction and immunosuppressive activity and it is an important local and systemic immunomodulator by placental exosome association (Lokossou et al., 2020). The major facilitator superfamily domain containing 2A (MFSD2A) is a homologous receptor for syncytin-2-mediated cell-cell fusion. Both syncytin-2 and MFSD2A are highly expressed in the placenta. The expression of syncytin-2 and MFSD2A in placental cells is regulated by the placental transcription factor and glial cells missing 1 (GCM1). In addition to playing an important role in placenta formation, syncytin-2 also plays a role in osteoclast and macrophage fusion. However, under experimental conditions, it is not essential for osteoclast and foreign body giant cell (FBGC) formation or bone homeostasis maintenance *in vivo* (Coudert et al., 2019).

The expression of syncytin-2 was associated with the progression of cancer and syncytin-2 was significantly over-expressed in pT2 endometrial carcinomas compared to pT1b endometrial carcinomas (Strissel et al., 2012). In chemo-resistant glioblastoma cells, cytotoxic stress promotes accumulation and fission of mitochondria, and the expression of syncytin-1 and syncytin-2 (Díaz-Carballo et al., 2017).

#### Glial Cell Missing 1 is Involved in Cell Fusion by Syncytin-1 Expression

Recent studies have shown that GCM1 is involved in syncytin-1 expression during embryonic development, and it may also be the regulatory center of the syncytin-1 signaling pathway (Chiu and Chen, 2016; Lu et al., 2016; Lu et al., 2017). GCM1 is an embryo-specific transcription factor containing a zinc finger structure, which is highly expressed in placental trophoblast cells. Its main role is to regulate the expression of syncytin-1 and mediate the formation of multinuclear syncytiotrophoblasts from mononuclear cell trophoblasts (Yu et al., 2002; Liang et al., 2010). Its production in the placenta is followed by placental differentiation processes, such as intercellular gap junction formation, cell syncytialization, and an increase in β-human chorionic gonadotropin (β-HCG) secretion. Some studies have found that overexpression of GCM1 can increase syncytin-1 expression and promote mutual fusion between BeWo cells. In addition, studies have shown that after silencing GCM1 by RNA interference or antisense oligonucleotides, cell fusion of BeWo cells is inhibited by preventing the formation of a syncytiotrophoblast layer (Baczyk et al., 2009). In another study, a GCM1 gene knockout in mice led to failure in the formation of the mesotrophoblast layer of the placenta, and a loss of the fusion ability to form syncytiotrophoblast cells, resulting in placenta formation failure (Chang et al., 2005). The exact mechanism of GCM1’s involvement in the regulation of syncytin-1 expression may be that GCM1 can recognize two GCM1 binding sites in the 5′-long terminal repeat (5′-LTR) region upstream of the syncytin-1 gene, activate the gene, and increase the expression of the syncytin-1 pro-fusion protein to enhance cell fusion (Lin et al., 2005). Ectopic expression of GCM1 can also activate the expression of syncytin-2 and MFSD2A in MCF-7 breast cancer cells and promote cell fusion. GCM1 may also play an important role in epigenetic regulation of syncytin-2 gene expression (Liang et al., 2010). In human trophoblast cells, neuropeptide FF (NPFF) binds to NPFF receptor 2 (NPFFR2) and promotes GCM1-dependent syncytin-1 and 2 expression (Zhu et al., 2020). In mice, GCM1 blocks mitosis and is required for syncytiotrophoblast formation and morphogenesis of the labyrinth, which is the murine equivalent of the villous placenta. GCM1 also plays a distinct role in the maintenance, development, and turnover of human trophoblasts.

#### Galectin-1 is Involved in Cell Fusion Associated With Syncytin-2 Expression

Galectin (Gal)-1, a soluble lectin, is also involved in trophoblast cell fusion. Studies have shown that Gal-1 has a specific and significant effect on syncytin-2 pseudovirus infection, which depends on the expression of MFSD2A. In addition, another placental lectin, Gal-3, does not modulate the infectivity of syncytin-2-positive viruses, reinforcing the specific association between Gal-1 and syncytin-2. Gal-1 significantly reduced the infectivity of a syncytin-1 pseudovirus, suggesting that Gal-1 had opposite effects on syncytin-1 and syncytin-2. Therefore, it is speculated that Gal-1 specifically interacts with syncytin-2 and may regulate syncytin-2/MFSD2A interactions during trophoblast syncytialization (Toudic et al., 2019).

#### Other Proteins Involved in Cell Fusion

During fertilization, oocyte tetrosomal proteins CD9 and CD81 are critical to the fusion event (Kaji et al., 2000; Miyado et al., 2000), while IZUMO and A disintegrins and metalloproteinase (ADAM) sperm proteins (Evans, 2001; Inoue et al., 2005) also participate in the fusion process. Glucose-regulatory protein 78 kDa (GRP78) is an endoplasmic reticulum protein that promotes cell fusion and exists on the surface of trophoblast cells (Ren et al., 2018; Bastida-Ruiz et al., 2020). GRP78 can also be expressed on the surface of cancer cells (Taghizadeh et al., 2021) and may play a role in cancer cell fusion. In myoblasts, the immunoglobulin (Ig) class of cell adhesion molecules is essential for cell-cell recognition and fusion (Ben-Zvi and Volk, 2019; Lee and Chen, 2019). In osteoclasts, CD47, CD200, dendritic cell-specific transmembrane protein (DC-STAMP), and osteoclast-stimulating transmembrane protein (OC-STAMP) are important for macrophage fusion (Miyamoto, 2011; Miyamoto et al., 2012; Wang et al., 2019; Zou et al., 2021). E-cadherin, cadherin-11, zona occludens-1 (ZO-1), and conjunctin-43 are also involved in cell-cell fusion (Getsios and MacCalman, 2003; Aghababaei et al., 2015; Gerbaud and Pidoux, 2015).

Myomaker (Tmem8c) is a muscle-specific protein which is necessary for myoblast fusion, and present on the plasma membrane (Gamage et al., 2017). Myomixer localizes to the plasma membrane, where it promotes myoblast fusion and associates with Myomaker, its expression coincides with myoblast differentiation and is essential for fusion and skeletal muscle formation during embryogenesis (Bi et al., 2017). Quinn, M. E., et al. show that Gm7325, which they name myomerger, induces the fusion of myomaker-expressing fibroblasts (Quinn et al., 2017). Annexins are composed of 12 members that can bind to membranes. Annexins molecule has multiple Ca^2+^-binding sites and calcium may mediate the fusion reaction, or induce a conformational change in a fusogenic protein (Papahadjopoulos et al., 1990). Annexin A1 and Annexin A5 are important for myoblast fusion. Endogenous Annexin A1 co-localizes with actin fibers at the ends of undifferentiated cells and involved in the cell fusion. Annexin A5 combined with a molecular complex including E-Cadherin, alpha-catenin and beta-catenin participate in the cell fusion (Degrelle et al., 2017). In addition, Yang et al. (2017) confirmed that spectraplakin/VAB-10A is an actin-binding protein that can bind to *Caenorhabditis elegans* fusogen EFF-1, which promoted cell-cell fusion in their experimental studies. Mutations in EFF-1 or VAB-10A attenuated actin dynamics in the cortex. They expounded cell-cell fusion as a positive feedback regulation process, in which fusogens are recruited to fusion sites by actin filaments that have been crosslinked by spectraplakin to gather more fusogens to form fusion synapses. Interleukin-4 (IL-4), receptor activator of nuclear factor-kappa B ligand (RANKL), matrix metallopeptidase 9 (MMP-9), E-cadherin, CD200, DC-STAMP, OC-STAMP, CD44, and purinergic type 2 receptor 7 (P2X7) have all been shown to play a role in macrophage fusion (Dörnen et al., 2020a). Influenza hemagglutinin (HA) is a viral membrane protein responsible for the initial entry of influenza virus into host cells. It mediates the binding of virus particles to host cell membranes and catalyzes the fusion of virus membranes with host cell membranes (Boonstra et al., 2018).

### Cell Fusion Related Signaling Pathways

Multiple signaling pathways have been found to regulate syncytin-1 expression and affect cell fusion. During embryonic development, syncytin-1 expression is regulated by multiple signaling pathways, such as the cyclic adenosine-dependent protein kinase A (cAMP/PKA), mitogen-activated protein kinase (MAPK), wingless/integrase-1 (Wnt), and c-Jun N-terminal kinase (JNK) signaling pathways (Knerr et al., 2005; Strick et al., 2006; Matsuura et al., 2011). Matsuura et al. (2011) confirmed that the β-catenin/B-cell CLL/lymphoma 9-like protein (BCL9L)/T-cell factor 4 (TCF4) signaling pathway targets the GCM1/syncytin pathway and regulates cell fusion *in vivo* and *in vitro*. A signal transduction pathway that links Wnt/β-catenin signaling and cell fusion in mammalian cells was also demonstrated. The amounts of β-catenin, BCL9L, and the modified active form of TCF4 in the nuclear fraction increased significantly in response to forskolin (FK) treatment. Other studies have shown that siRNA-mediated knockdown of BCL9L, β-catenin, and TCF4 in FK-treated BeWo cells resulted in a significant downregulation of FK-induced expression of GCM1, syncytin-1, syncytin-2, and axis inhibition protein 2 (AXIN2) (Suzuki et al., 2016; Huge et al., 2020). The expression pattern of BCL9L in the chorion coincided with those of GCM1 and syncytin-2, which is consistent with the idea that BCL9L is an upstream regulator of the GCM1/syncytin pathway. In addition, Knerr et al. (2005) demonstrated that the PKA pathway is upstream of GCM1. After transient transfection of BeWo cells with PKA, GCM1 transcriptional activity and GCM1 and syncytin transcripts were upregulated. In addition to activation and stabilization of GCM1 through PKA phosphorylation, PKA may also activate cofactors, such as CREM binding protein (CBP), through phosphorylation. Previously, CBP has been shown to act jointly with proteins, such as cAMP responsive element modulator (CREM)/cAMP response element binding protein (CREB)/activating transcription factor 1 (ATF1) or NFκB, and it might also be able to associate, and thereby modulate, the DNA binding capacity of GCM1. Binding motifs for CREM and CREB were shown to be localized to the GCM1 promoter. The study demonstrated that hypoxia-related downregulation of syncytin transcription in trophoblasts can be, to a great extent, compensated by stimulating the cAMP-driven PKA pathway. Strick et al. (2006) reported that syncytin-1 was significantly increased at the mRNA and protein levels in endometrial carcinomas compared to controls. Activation of the cAMP pathway resulted in syncytin-1 upregulation, and cell fusions similar to placental syncytiotrophoblasts occurred. TGF-β1 and TGF-β3 are major negative regulators of cell fusion, especially in steroid-induced cell proliferation of syncytin-1 in endometrial carcinomas. TGF-β1 and TGF-β3 inhibited cell fusion, whereas antibody-mediated TGF-β neutralization induced cell fusions, indicating that TGF-β induction could override syncytin-1-mediated cell fusions.

### Cell Fusion in Embryonic Development

**FIGURE 2:**
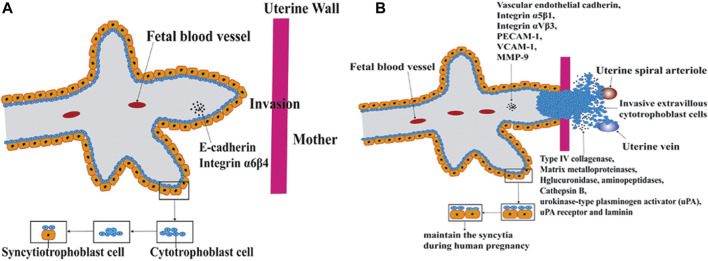
Cell fusion in placenta formation. The formation of the placenta is accompanied by cell fusion and is divided into two stages according to the different roles in the fusion process. In the first phase, cytotrophoblast cells fuse to form polynuclear syncytiotrophoblast cells. In the second phase, cytotrophoblasts and syncytiotrophoblasts fuse for tissue renewal. **(A)** Cytotrophoblast cells fuse to form polynuclear syncytiotrophoblast cells. **(B)** cytotrophoblasts and syncytiotrophoblasts fuse for tissue renewal.

Sperm/egg fertilization events during sexual reproduction, syncytiotrophoblast formation in the placenta occur with the process of cell fusion (Petrany and Millay, 2019). Gamete fusion occurs at a developmental stage in humans. During gamete fusion, some proteins are directly involved in the core fusion process. Valansi et al. (2017) demonstrated that loss of HAP2/GCS1 proteins resulted in gamete fusion failure. Sperm IZUMO proteins may play a role in fertilization by regulating cell fusion. The loss of the IZUMO protein in male mice results in sterility due to sperm-egg fusion failure, even if the sperms are normal in shape and mobility and able to cross the zona pellucida successfully (Inoue et al., 2005). Saito et al. (2019) confirmed that the expression level of the IZUMO1 protein in mice was positively correlated with fertility. Other proteins may play a part before fusion, promoting communication and adhesion between cells, such as CD9, Juno, and IZUMO receptors, which have been reported to play a similar role (Bianchi et al., 2014). The formation of the placenta is accompanied by cell fusion and is divided into two stages according to the different roles in the fusion process (Figure 2). In the first phase, cytotrophoblast cells fuse to form polynuclear syncytiotrophoblast cells, which migrate and invade the mother’s uterus and serve as an exchange channel for oxygen, nutrients, and metabolic wastes between the embryo and the mother. Interestingly, syncytiotrophoblasts are formed with the ability to migrate and invade like cancer cells at this stage. In the second phase, cytotrophoblasts and syncytiotrophoblasts fuse for tissue renewal (Gerbaud and Pidoux, 2015).

## Cell Fusion in Cancer

**FIGURE 3:**
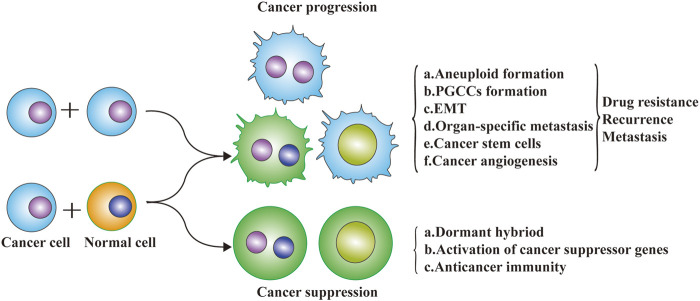
Cell fusion and cancer progression. Cell fusion is a two-edge sword that occurs during the development and progression of cancer. The exchange of DNA between cancer cells and non-cancer cells *via* cell fusion may result in a different fate of cancer cells. Metastatic potential genes from macrophages (or cancer cells) to cancer cells may enhance the migration and invasive abilities. Tumor suppressor genes exchanged from normal cells to cancer cells may inhibit the malignant progression of cancer cells.

Cell fusion is a two-edge sword that occurs during the development and progression of cancer (Figure 3). Studies of the relationship between cell fusion and cancer date back to the 1900s when Otto Aichel hypothesized that spontaneous fusion of somatic cells could lead to chromosomal abnormalities and cancer (Lu and Kang, 2009a).

Cell fusion is involved in the initiation of cancer and participates in organ-specific metastasis (seed and soil hypothesis), epithelial-mesenchymal transformation, CSC formation, and cancer angiogenesis, which greatly enhances the migration and invasion capacity of cancer cells, leading to chemotherapy resistance, recurrence, and metastasis. Cell fusion not only changes the composition and biological characteristics of cancer cells, but also changes the cancer microenvironment to promote cancer development. Cell fusion contributes to the initiation of cancer, in which inflammation plays a major role because the tumor environment resembles chronic inflammatory tissue (Schmidt and Weber, 2006). Fusion between tumor cells and neighboring tumor cells or normal tumor-infiltrating cells may be a common process in cancer progression. Results of Powell, A. E. et al. proved that cell fusion between circulating blood-derived cells and tumor cells occurs during tumorigenesis, and the fusion between macrophages and tumor cells could impart metastatic potential to tumor cells (Powell et al., 2011). It has been proposed that the fusion of tumor cells and leukocytes might be related to distant metastasis of tumors, and some scholars believe that the fusion of multiple tumor cells might be closely related to tumor metastasis and tumor phenotypic diversity (Pawelek and Chakraborty, 2008). Spontaneous fusion of a human glioblastoma with normal hamster cells in animal xenografts can result in malignant cells that express both human and hamster genes, retain malignant tumor characteristics and have stronger metastatic ability than the parental cells (Goldenberg et al., 2012). In one report, after a renal cancer patient received hematopoietic stem cell transplantation, cell fusion was found between recipient tumor cells and donor hematopoietic stem cells in a metastatic lymph node tumor by comparing the immunophenotype of the patient and bone marrow transplantation donor (Chakraborty et al., 2004). In another case, a Y chromosome was found in the renal tumor cells of a woman who had received hematopoietic stem cells from a healthy man, which also demonstrated that the recipient’s renal cancer cells had fused with the donor’s hematopoietic stem cells.

However, cell fusion may also have the ability to prevent malignant transformation of tumors by potentially correcting genetic and/or phenotypic changes underlying malignant transformation. *p53* plays a critical role in regulating cell cycle arrest and apoptosis, and the growth of HepG2 can be inhibited by inducing apoptosis after transducing wild-type *p53* into cancer cells *in vitro* (Yan et al., 2012). Although spontaneous cell fusion that inhibits tumor development was not observed *in vivo*, in artificially induced normal cells and malignant tumor cell fusion experiments, hybrid cells no longer had the ability to differentiate into malignant tumor cells. In the process of integration, the existence of certain genes may protect normal cells from malignant transformation. As early as 1969, Henry Harris reported that the fusion of normal mouse fibroblasts with various malignant mouse cell lines resulted in the formation of hybrid cells with stable chromosomal markers from both parent lines, which did not form tumors in histocompatible mice (Harris et al., 1969). Malignant tumors do not spontaneously fuse with normal cells and lose their ability to become malignant (Platt et al., 2016). However, artificially-induced specific cell fusion can reverse malignant transformation and tumor progression. It has been suggested that cell fusion can be used as a tissue-level defense to inhibit the occurrence and development of malignant tumors (Platt and Cascalho, 2019a). A growing number of studies have found that tumor-associated non-malignant cells can play a role in the transformation and progression of cancer at the tissue level, including through contact inhibition, population maintenance, and the partitioning of stem cells and chromosomal DNA in ways that monopolize the accumulation of DNA copying errors (Werner and Sottoriva, 2018). Normal cells, such as fibroblasts, may also inhibit tumor growth and progression (Alkasalias et al., 2018). For example, breast cancer cells have been found to be likely to phagocytose and fuse with normal mesenchymal cells or fibroblasts. Results of Melzer C, et al. showed that the hybrid cells derived from the co-culture of SKOV-3 and MSCs can form two differentially fused ovarian cancer cell populations by RNA microarray analysis, and both ovarian cancer hybrid populations exhibited reduced proliferative capacity compared to the parental SKOV-3 cells. After fusion, the hybrid is inactive and appears to be dormant, and rapid proliferation, migration, and invasion temporarily ceases (Melzer et al., 2018). Tumor cells survive in a resting state that significantly reduces their ability to grow. Therefore, cell fusion can not only cause malignant transformation and progression of cancer cells, but it can also reduce malignancy into dormancy. Such differences should not only be determined by cell fusion alone, but should also be related to the tumor microenvironment and the overall environment of the human body.

### Heteroploidy by Cell Fusion and Carcinogenesis

**FIGURE 4:**
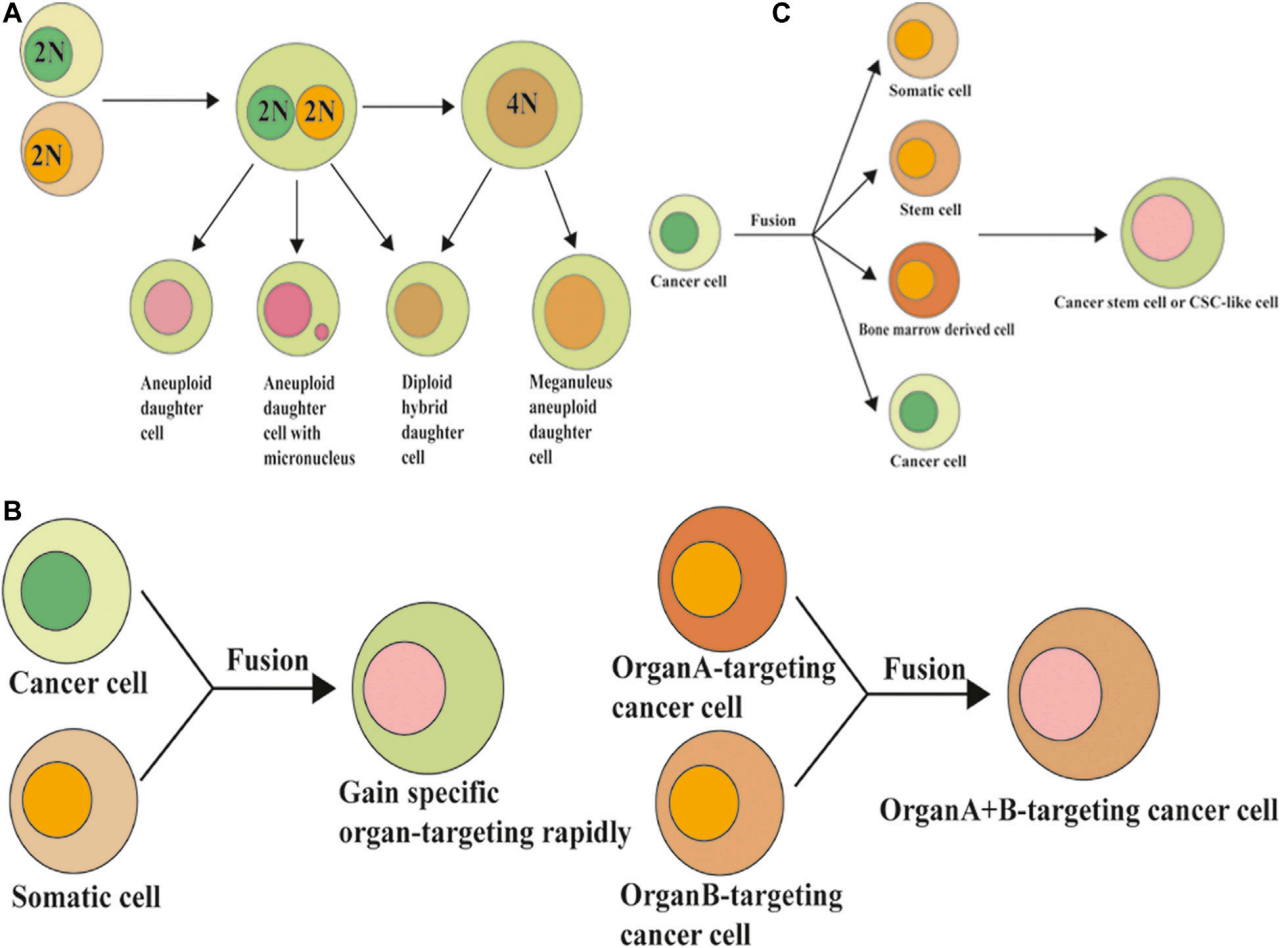
Cell fusion and the formation of polyploidy, organ-specific metastasis, and formation of cancer stem cells. **(A)** Cell fusion induced polyploidy, aneuploidy, and genomic instability. **(B)** Cell fusion and organ-specific metastasis of cancer cells. **(C)** Cell fusion and the formation of cancer stem cells.

Cancer cells may exhibit heterogeneity by fusion between themselves or with adjacent cells, such as stromal cells, epithelial cells, endothelial cells, and macrophages, which are the source of gene instability. This process also allows cancer cells to acquire metastatic and drug resistance abilities by enhancing heterogeneity, increasing proliferation, enhancing invasion, and strengthening their overall function to ensure survival (Bastida-Ruiz et al., 2016). The hybrid cells generated after cell fusion acquire the biological characteristics of both parent cells, thus increasing the polymorphism and heterogeneity of tumor cells, and enhancing the proliferation, invasion, metastasis, drug resistance, and anti-apoptosis of the progenitor cells. This occurrence directly or indirectly promotes distant metastasis of tumor cells (Duelli and Lazebnik, 2003; Shabo et al., 2020). The hybrid cells produced through heterotypic cell fusion not only possess features from both parental cells, but also exhibit stronger colony formation, proliferation, migration, and apoptosis inhibition abilities (Zhang et al., 2019a). Therefore, it is of great significance to explore the specific molecular mechanisms of cell fusion and to develop new therapeutic methods for cancer treatment. The cell fusion model holds that before a tumor cell can metastasize further, it must acquire the ability to multiply beyond infinity. In response to changes in the environment, cell fusion can produce hybrid cells through horizontal gene transfer (cell-to-cell gene transfer), which quickly acquire new genotypes by non-genetic mutations to adapt to changes in the environment. Therefore, as a genomic non-mutational mechanism, cell fusion can better explain chromosome aneuploidy, gene rearrangement, and abnormal gene expression seen in malignant tumor cells (Figure 4A).

Polyploidy is considered the transition stage from healthy diploid cells to tumor aneuploid cells, which can form aneuploid cells after cell division (Storchova and Pellman, 2004). Cell fusion is just one of the causes of polyploid formation. Other mechanisms, including cytokinesis failure, mitotic slippage, endoreplication, endomitosis, and abortive phagocytosis, could also contribute to polyploid formation (Orr-Weaver, 2015). Searles et al. (2018) found in their research that cancer cells can spontaneously and rapidly exchange DNA with non-cancer cells *via* fusion events in the natural progression of cancer *in vivo* or *in vitro*, leading to a large number of genomic changes. In their study, the hybrid syncytia formed by cancer cell and non-cancer cell fusions were aneuploid and had more clone diversity and chemotherapeutic resistance than non-hybrid cancer cells. The exchange of DNA between cancer cells and non-cancer cells *via* cell fusion may result in a different fate of cancer cells. Metastatic potential genes from macrophages to cancer cells may enhance the migration and invasive abilities. Tumor suppressor genes (such as wild-type *p53*) exchanged from normal cells to cancer cells may induce the apoptosis of cancer cells.

Yin et al. (2020) showed that clones of the hybrid progenies fused between the cancer and neural stem cells were highly heterogeneous with neuroendocrine features, with most of the progenies having acquired neural marker expression but having lost prostatic and epithelial markers. Pawelek (2005) concluded that tumor cells obtain myeloid traits by fusing with myeloid cells, which is a process that might also contribute to aneuploidy and plasticity in cancer. Other recent studies have also confirmed that tumor cells and neighboring tumor cells, leukocytes, macrophages, endothelial cells, lymphocytes, and other host cells can undergo this cell fusion phenomenon. Many experiments have found that polyploid and highly invasive progeny hybrid cells were produced by the fusion of malignant or benign tumor cells with bone marrow-derived cells (He et al., 2005).

As mentioned earlier, if two or more cells fuse together, they can either form heterokaryons containing multiple individual nuclei, or the nuclei may fuse to produce synkaryons. Both these conditions can occur in human tumors (Dittmar et al., 2011). At the same time, binuclear and multinucleated hybrid cells can also undergo ploidy reduction/heterokaryon-to-synkaryon transition (HST), resulting in mononuclear and binuclear cells (Bjerkvig et al., 2005; Duncan et al., 2009; Sottile et al., 2016; Frade et al., 2019; Matsumoto et al., 2020). The reduced ploidy/HST of hybrid cells can produce both normal diploid karyotype daughter cells (Skinner et al., 2012) and genomically unstable aneuploid daughter cells (Pellman, 2007; Dürrbaum and Storchová, 2016; Chunduri and Storchová, 2019).

### Cell Fusion and Polyploid Giant Cancer Cells

PGCCs are a special sub-population of cancer cells that were previously considered to be senescent cells. Recent studies have confirmed that PGCCs possess CSC properties, with the expression of the CSC markers CD44 and CD133. Fused cancer cells can contribute to the formation of PGCCs, which are highly tumorigenic and chemoresistant (Zhang et al., 2014a; Was et al., 2021). PGCCs exhibiting CSC-like properties can be induced by various stimuli, including hypoxia, chemotherapy, and radiotherapy, and contribute to cellular heterogeneity, stemness, chemoresistance, metastasis, and tumor progression (Zhang et al., 2014a; Zhang et al., 2014b; Zhang et al., 2017). Li et al. reported that cell fusion was observed during the formation of PGCCs *via* the GCM1/syncytin-1 signaling pathway. Clinically, the expression of cell fusion-related and erythroid differentiation-related proteins gradually increases with the progression of human colorectal cancer tissues (Li et al., 2021).

### Cell Fusion and Epithelial-Mesenchymal Transformation

EMT is an important marker of tumor invasion and metastasis, and the loss of an epidermal phenotype and gain of a mesenchymal phenotype are the main characteristics (Jolly et al., 2019; Katsuno and Derynck, 2021). During EMT, epithelial cells lose their epithelial characteristics, such as polarity and intercellular adhesion, and transform into mesenchymal cells with high invasion, migration, anti-apoptosis, and extracellular matrix degradation abilities. Therefore, tumor cells promote cell migration, motility, invasion, and metastasis *via* EMT (Liu et al., 2021).

Recent studies have shown that when tumor cells fuse with somatic or mesenchymal stem cells, the EMT characteristics of the fused cells are stronger than those of the parental cells. Various cases of tumor cell and normal cell fusion have been reported in the literature, such as human gastric cancer cells (HGC-27 and SJC-7901) (Xue et al., 2015), human hepatoma cells (HepG2) (Li et al., 2014), human breast cancer cells, and human lung cancer cells (A549, H460, etc.) fusing with human umbilical vein mesenchymal stem cells (Xu et al., 2014), and human endometrial cancer cells fusing with stromal cells (Li et al., 2019). The hybrid progenies produced after cell fusion developed the EMT phenomenon and had stronger invasion, metastasis, and tumorigenesis abilities than the parental cells (Xue et al., 2015; Dörnen et al., 2020b). When normal human gastric mucosa cells (GES-1) were fused with hematopoietic stem cells, it was found that the fused cells not only underwent EMT, but also showed malignant transformation of normal gastric mucosa epithelial cells (He et al., 2015). It has also been reported that human breast cancer cells can fuse with endothelial cells, allowing the tumor cells to cross the endothelial barrier, thereby promoting metastasis (Mortensen et al., 2004).

### Cell Fusion and Organ-Specific Metastasis of Cancer Cells

Distant metastases of solid tumors are organ-specific, such as the tendency for colorectal cancer to metastasize to the liver, while prostate cancer tends to metastasize to the bone (Lu and Kang, 2009b; Radeczky et al., 2021; Waza et al., 2021). Currently, the seed-soil theory is the most widely accepted theory of tumor metastasis. The theory suggests that different tumors metastasize to different specific organs, and that this targeted metastasis is due to the selection of “seeds” (tumor cells) for “soil” (metastasis target organs). Recent studies have found that cell fusion is closely related to the organ orientation of metastatic tumors. Once the tumor cells fuse with the intrinsic cells of the target organ, the tumor cells may acquire the characteristics of the target cells and successfully escape immune rejection by the host cells to survive, resulting in extensive metastasis (Lu and Kang, 2009a). Moreover, fused cells can also participate in the remodeling of the target organ microenvironment, making the microenvironment conducive to the occurrence, development, and metastasis of tumors (Song et al., 2012).

The fusion of tumor cells and macrophages plays an important role in the enhancement of tumor invasion ability and the tendency to metastasize to other organs (Figure 4B). It has been reported that heterozygous cells from the fusion of myeloma with macrophages tend to metastasize to the lungs, while heterozygous cells from the fusion of myeloma with B lymphocytes tend to metastasize to the liver and spleen (De Baetselier et al., 1984). It has also been reported that the progenitors of melanomas that fuse with macrophages are more likely to migrate to the lung. As a cell phenotype, the organ propensity of tumor metastasis can also be rapidly transmitted to progenitor cells through cell fusion, and these malignant features can be stably expressed in progenitor cells after long-term passage *in vivo* and *in vitro*. Therefore, cell fusion may be a way for tumor cells to rapidly acquire organ specificity for metastasis through horizontal gene transmission (Chakraborty et al., 2000). In conclusion, the cell fusion hypothesis perfectly explains the “seed-soil” theory and organ-targeting of solid tumor metastasis.

### Cell fusion and the Formation of Cancer Stem Cells

CSCs are a type of cancer cell subset with the characteristics of stem cells that have the ability to self-renew and differentiate into various cancer cells. CSCs are the source of cancer recurrence and metastasis and are the basis of long-term survival and progression of cancer (Fu et al., 2017). Cell fusion may be one of the mechanisms by which CSCs are produced. Many studies have found that tumor cell-somatic cell, tumor cell-stem cell, tumor cell-bone marrow derived cells (BMDSCs), and other mutual fusions may be one of the sources of CSCs (Figure 4C). Several studies have shown that BMDSCs can fuse with somatic or tumor cells to form CSCs. BMDSCs can spontaneously fuse with tumor cells to generate hybrid cells with high proliferation and metastatic capacity, thus participating in tumor initiation and progression (Pawelek, 2005).

Gauck et al. demonstrated that the spontaneous fusion of human breast cancer cells and breast epithelial cells can produce hybrid cells with CSC characteristics, which have stronger colony formation ability (Gauck et al., 2017). In lymph node metastasis of breast cancer, the expression of breast stem cell markers CD44+/CD24- and stromal marker vimentin are increased, and epithelial marker E-cadherin is decreased. Therefore, after cell fusion, the progenitor tumor cells can not only develop EMT and become highly invasive, but also acquire the characteristics of CSCs (Hass et al., 2019). Zhang et al. (2019b) found that heterotypic hybrid cells formed by lung cancer cells and mesenchymal stem cells spontaneously expressed stem cell marker progenitor-1 30 times higher than their parent lung cancer cells. Expression of stem cell phenotype-related proteins, such as B-lymphoma Mo-MLV insertion region 1 (Bmi-1), transcription factor octamer-binding transcription factor 4 (Oct-4), and sex determining region Y-Box 2 (Sox2), were also increased in the hybrid cells. After the fusion of gastric cancer cells and mesenchymal stem cells, the expression of epithelial markers, such as E-cadherin, in their progenies decreased, while the expression of mesenchymal markers, such as vimentin and N-cadherin, increased. Meanwhile, the expression levels of tumor stem cell markers, such as Oct4, Sox2, Lin28, Nanog homeobox, CD133, and CD44, in progenitor cells also increased (Xue et al., 2015). Therefore, cell fusion may lead to progenitor cells possessing both CSC and EMT properties, thus making progenitor cells more capable of invasion and metastasis. LaBerge et al. reported that malignant mononuclear cells isolated from a melanoma in a bone marrow transplant recipient where the melanoma had metastasized contained DNA from the bone marrow transplant donor and the recipient, suggesting that there is a high probability of spontaneous fusion between recipient and donor cells *in vivo* (LaBerge et al., 2017).

Cancer drug resistance is a major challenge in cancer treatment and is often explained by two models. The first model, which is similar to the theory of biological evolution, is that cancer cells adapt to the microenvironment and acquire different phenotypes. In the presence of therapeutic drugs, a large number of cancer cells die, while a small number of resistant cells survive and remain hidden and may go undetected in routine tests. The second model suggests that the presence of CSCs allow cancer cells to survive the chemotherapeutic drug. Both models can be explained by cell fusion (Valcz et al., 2020). Studies have confirmed that the tumorigenic subpopulation of mouse leucine-rich repeat-containing G-protein coupled receptor 5 (LGR5)-positive cells exists in a slow-cycling state and a unique 22-gene signature that characterizes these slow-cycling CSC-like cells, which often contributes to cancer chemoresistance, has been identified (Shiokawa et al., 2020). Other studies have shown that the presence of CSC-like cells can help tumor cells avoid the lethal effects of radiation, chemotherapy, and other treatments.

Fusion of cancer cells can form CSCs, enabling cancer cells to survive even in an environment that is not conducive to their survival, which increases tumorigenicity, invasiveness, and metastatic potential (Zhang et al., 2014a). Fusion of CSCs can enable cells to differentiate into various cell types and plays a role in promoting tumor cells in the body; for example, tumor ectomesenchymal cells, endothelial cells, and fibroblasts can secrete growth factors, create a favorable microenvironment for tumor survival, support the expansion of tumor volume, induce extracellular matrix concentrated growth factor secretion, and support tumor cell proliferation. The inherent non-malignant cells of the tumor also help to inhibit or block external defenses, such as tumor immunity. The nutritional roles of these cells, such as activated fibroblasts, have been extensively explored and elucidated (Alkasalias et al., 2018; Poltavets et al., 2018).

### Cell Fusion and Cancer Angiogenesis

The occurrence, development, invasion, and metastasis of malignant tumors depend on tumor angiogenesis, but the mechanism of tumor angiogenesis remains unclear. At present, it is generally believed that tumor cells provide blood supply to tumors mainly through endothelial cell-dependent blood vessels, mosaic blood vessels, and vascular mimicry (VM). It has been reported that the fusion of tumor cells and host cells (such as white blood cells and macrophages), CSCs, and mesenchymal stem cells can promote vascular proliferation, thus promoting tumor invasion and metastasis.

After the fusion of tumor cells with bone marrow-derived cells, the progenitor tumor cells acquire the characteristics of the bone marrow-derived cells, with increased angiogenesis and cell activity, which may also be an important reason for tumor development and metastasis. Bone marrow mesenchymal stem cells and glioma stem cells can also drive tumor angiogenesis through cell fusion (Sun et al., 2019). In addition, one study showed that the microvascular density and vascular maturity of the heterozygous generation of macrophage and sarcoma cells were higher than those of the parental cells, and the expression levels of VEGF and TGF-β in the progenitor cells were also higher than those in the parental tumor cells (Busund et al., 2002; Shen et al., 2015).

Cell fusion may play an important role in cancer angiogenesis by promoting CSC-like cells (PGCCs) generation. PGCCs may directly promote tumor angiogenesis through transdifferentiation and may also participate in VM formation. PGCCs with their erythroid progeny can form VM structures to facilitate tumor growth (Zhang et al., 2014c; Yang et al., 2018). VM can interact with endothelial cell-dependent channels to provide blood and oxygen for tumor growth, invasion, and metastasis (Zhang et al., 2015; Zhang et al., 2017; Yang et al., 2018). Recently, Li, et al. reported that PGCCs can generate red blood cells and form VMs (Zhang et al., 2015; Li et al., 2021). In addition, the red blood cells produced by PGCCs have the characteristics of embryonic and fetal hemoglobin. Hemoglobin has a high binding force and affinity for oxygen, which can meet the oxygen needs of tumor cells under severe hypoxia and provide sufficient energy and oxygen for tumor invasion and metastasis (Zhang et al., 2014c). Therefore, under the action of various inducers, tumor cells can form PGCCs with the characteristics of CSCs through cell fusion, differentiate into hematopoietic cells, and form VM (Hassan and Seno, 2020).

### Cell Fusion and Cancer Immunity

The cancer immune editing model imagines the dynamic relationship between the immune system and cancer. In this model, many problems are difficult to explain, while some problems can be easily explained by introducing the concept of cell fusion. The whole process includes three stages. In the first stage, malignant transformation may be due to mutation and recombination, forming tumors with partial immunogenicity and diversity. In the second stage, which is the stage of tumor screening, the immune system and the tumor adapt to each other. Cells that have acquired resistance to cytotoxicity and immune attack accumulate and survive. In the third stage, the tumor progresses significantly, becomes more diversified and adaptable, escapes immune surveillance and killing, and continues to grow (Schumacher and Schreiber, 2015; Platt and Cascalho, 2019b). The mechanism of clonal diversification and antigen presentation in this model can also be understood by introducing cell fusion. Cell fusion can rapidly allow multiple chromosomal changes and mutations to occur simultaneously, and multiple malignant clones may remain genetically stable long enough to produce sufficient numbers of neoantigens to stimulate immunity before further diversification occurs. Fusion of malignant cells also produces auxiliary signals required to recruit and activate antigen-presenting cells (APCs). Most importantly, the mutant cells fuse with dendritic cells or other APCs to produce a strong and protective immunity (Koido, 2016).

## Conclusion

Although cell fusion adapts to the needs of different environmental changes and occurs in response to different inducing factors, it is believed that cell fusion in cancer may have regulatory mechanisms similar to those of other physiological cell fusion processes (Blumenthal et al., 2003; Wickner and Schekman, 2008). Cell fusion mediated by the proteins and pathways discussed in this review plays an important role in physiological processes, such as placental development, but the role of cancer cell fusion is still convoluted and needs further investigation.

## Glossary

CSCs: cancer stem cells
PGCCs: polyploid giant cancer cells
EMT: epithelial-mesenchymal transformation
HAP2/GCS1: hapless 2/generative cell-specific protein 1
T-SNARE: target-soluble N-ethylmaleimide-sensitive factor attachment protein receptor
V-SNARE: vesicular-SNARE
HERV: human endogenous retrovirus
SU: surface subunit
TM: transmembrane subunit
ASCT-2: sodium-dependent type 2 neutral amino acid transporter
SPRE: post-transcriptional regulatory element
MFSD2A: major facilitator superfamily domain containing 2A
GCM1: glial cells missing 1
NPFF: neuropeptide FF
Gal: Galectin
GRP78: glucose-regulatory protein 78 kDa
DC-STAMP: dendritic cell-specific transmembrane protein
OC-STAMP: osteoclast-stimulating transmembrane protein
cAMP/PKA: cyclic adenosine-dependent protein kinase A
Wnt: wingless/integrase-1
BCL9L: B-cell CLL/lymphoma 9-like protein
TCF4: T-cell factor 4
FK: Forskolin
CBP: CREM binding protein
CREM: cAMP responsive element modulator
CREB: cAMP response element binding protein
HST: heterokaryon-to-synkaryon transition
BMDSCs: bone marrow derived cells
Sox2: sex determining region Y-Box 2
Oct-4: octamer-binding transcription factor 4
VM: vascular mimicry
APCs: antigen-presenting cells

## References

[B1] Aghababaei M. Hogg K. Perdu S. Robinson W. P. Beristain A. G. (2015). ADAM12-directed Ectodomain Shedding of E-Cadherin Potentiates Trophoblast Fusion. Cell Death Differ 22 (12), 1970–1984. 10.1038/cdd.2015.44 25909890 PMC4816105

[B2] Alkasalias T. Moyano-Galceran L. Arsenian-Henriksson M. Lehti K. (2018). Fibroblasts in the Tumor Microenvironment: Shield or Spear? Ijms 19 (5), 1532. 10.3390/ijms19051532 PMC5983719 29883428

[B3] Baczyk D. Drewlo S. Proctor L. Dunk C. Lye S. Kingdom J. (2009). Glial Cell Missing-1 Transcription Factor Is Required for the Differentiation of the Human Trophoblast. Cell Death Differ 16 (5), 719–727. 10.1038/cdd.2009.1 19219068

[B4] Bastida-Ruiz D. Van Hoesen K. Cohen M. (2016). The Dark Side of Cell Fusion. Ijms 17 (5), 638. 10.3390/ijms17050638 PMC4881464 27136533

[B5] Bastida-Ruiz D. Wuillemin C. Pederencino A. Yaron M. Martinez de Tejada B. Pizzo S. V. (2020). Activated α2-macroglobulin Binding to Cell Surface GRP78 Induces Trophoblastic Cell Fusion. Sci. Rep. 10 (1), 9666. 10.1038/s41598-020-66554-0 32541810 PMC7295802

[B6] Ben-Zvi D. S. Volk T. (2019). Escort Cell Encapsulation of Drosophila Germline Cells Is Maintained by Irre Cell Recognition Module Proteins. recognition module proteins 8 (3), bio039842. 10.1242/bio.039842 PMC6451344 30837217

[B7] Bi P. Ramirez-Martinez A. Li H. Cannavino J. McAnally J. R. Shelton J. M. (2017). Control of Muscle Formation by the Fusogenic Micropeptide Myomixer. Science 356 (6335), 323–327. 10.1126/science.aam9361 28386024 PMC5502127

[B8] Bianchi E. Doe B. Goulding D. Wright G. J. (2014). Juno Is the Egg Izumo Receptor and Is Essential for Mammalian Fertilization. Nature 508 (7497), 483–487. 10.1038/nature13203 24739963 PMC3998876

[B9] Bjerkvig R. Tysnes B. B. Aboody K. S. Najbauer J. Terzis A. J. A. (2005). Opinion: the Origin of the Cancer Stem Cell: Current Controversies and New Insights. Nat. Rev. Cancer 5 (11), 899–904. 10.1038/nrc1740 16327766

[B10] Bjerregaard B. Holck S. Christensen I. J. Larsson L.-I. (2006). Syncytin Is Involved in Breast Cancer-Endothelial Cell Fusions. Cell. Mol. Life Sci. 63 (16), 1906–1911. 10.1007/s00018-006-6201-9 16871371 PMC11136146

[B11] Bjerregard B. Ziomkiewicz I. Schulz A. Larsson L.-I. (2014). Syncytin-1 in Differentiating Human Myoblasts: Relationship to Caveolin-3 and Myogenin. Cell Tissue Res 357 (1), 355–362. 10.1007/s00441-014-1930-9 24902667

[B12] Blaise S. de Parseval N. Heidmann T. (2005). Functional Characterization of Two Newly Identified Human Endogenous Retrovirus Coding Envelope Genes. Retrovirology 2, 19. 10.1186/1742-4690-2-19 15766379 PMC555746

[B13] Blumenthal R. Clague M. J. Durell S. R. Epand R. M. (2003). Membrane Fusion. Chem. Rev. 103 (1), 53–70. 10.1021/cr000036+ 12517181

[B14] Boonstra S. Blijleven J. S. Roos W. H. Onck P. R. van der Giessen E. van Oijen A. M. (2018). Hemagglutinin-Mediated Membrane Fusion: A Biophysical Perspective. Annu. Rev. Biophys. 47, 153–173. 10.1146/annurev-biophys-070317-033018 29494252

[B15] Brukman N. G. Uygur B. Podbilewicz B. Chernomordik L. V. (2019). How Cells Fuse. J. Cell Biol 218 (5), 1436–1451. 10.1083/jcb.201901017 30936162 PMC6504885

[B16] Busund L.-T. R. Killie M. K. Bartnes K. Seljelid R. (2002). Spontaneously Formed Tumorigenic Hybrids of Meth A Sarcoma and Macrophages Grow Faster and Are Better Vascularized Than the Parental Tumor. Int. J. Cancer 100 (4), 407–413. 10.1002/ijc.10502 12115521

[B17] Cáceres M. Thomas J. W. (2006). The Gene of Retroviral Origin Syncytin 1 Is Specific to Hominoids and Is Inactive in Old World Monkeys. J. Hered. 97 (2), 100–106. 10.1093/jhered/esj011 16424151

[B18] Chakraborty A. K. Sodi S. Rachkovsky M. Kolesnikova N. Platt J. T. Bolognia J. L. (2000). A Spontaneous Murine Melanoma Lung Metastasis Comprised of Host X Tumor Hybrids. Cancer Res. 60 (9), 2512–2519. Published May 2000 10811133

[B19] Chakraborty A. Lazova R. Davies S. Bäckvall H. Ponten F. Brash D. (2004). Donor DNA in a Renal Cell Carcinoma Metastasis from a Bone Marrow Transplant Recipient. Bone Marrow Transpl. 34 (2), 183–186. 10.1038/sj.bmt.1704547 15195072

[B20] Chang C.-W. Chuang H.-C. Yu C. Yao T.-P. Chen H. (2005). Stimulation of GCMa Transcriptional Activity by Cyclic AMP/protein Kinase A Signaling Is Attributed to CBP-Mediated Acetylation of GCMa. Mol. Cell Biol 25 (19), 8401–8414. 10.1128/mcb.25.19.8401-8414.2005 16166624 PMC1265739

[B21] Chen E. H. Olson E. N. (2005). Unveiling the Mechanisms of Cell-Cell Fusion. Science 308 (5720), 369–373. 10.1126/science.1104799 15831748

[B22] Cheynet V. Ruggieri A. Oriol G. Blond J.-L. Boson B. Vachot L. (2005). Synthesis, Assembly, and Processing of the Env ERVWE1/syncytin Human Endogenous Retroviral Envelope. J. Virol. 79 (9), 5585–5593. 10.1128/jvi.79.9.5585-5593.2005 15827173 PMC1082723

[B23] Chiu Y. H. Chen H. (2016). GATA3 Inhibits GCM1 Activity and Trophoblast Cell Invasion. Sci. Rep. 6, 21630. 10.1038/srep21630 26899996 PMC4761948

[B24] Chunduri N. K. Storchová Z. (2019). The Diverse Consequences of Aneuploidy. Nat. Cell Biol 21 (1), 54–62. 10.1038/s41556-018-0243-8 30602769

[B25] Coudert A. E. Redelsperger F. Chabbi-Achengli Y. Vernochet C. Marty C. Decrouy X. (2019). Role of the Captured Retroviral Envelope Syncytin-B Gene in the Fusion of Osteoclast and Giant Cell Precursors and in Bone Resorption, Analyzed *Ex Vivo* and *In Vivo* in Syncytin-B Knockout Mice. Bone Rep. 11, 100214. 10.1016/j.bonr.2019.100214 31360740 PMC6637224

[B26] De Baetselier P. Roos E. Brys L. Remels L. Gobert M. Dekegel D. (1984). Nonmetastatic Tumor Cells Acquire Metastatic Properties Following Somatic Hybridization with normal Cells. Cancer Metast Rev. 3 (1), 5–24. 10.1007/bf00047690 6370419

[B27] Degrelle S. A. Gerbaud P. Leconte L. Ferreira F. Pidoux G. (2017). Annexin-A5 Organized in 2D-Network at the Plasmalemma Eases Human Trophoblast Fusion. Sci. Rep. 7, 42173. 10.1038/srep42173 28176826 PMC5297248

[B28] Díaz-Carballo D. Klein J. Acikelli A. H. Wilk C. Saka S. Jastrow H. (2017). Cytotoxic Stress Induces Transfer of Mitochondria-Associated Human Endogenous Retroviral RNA and Proteins between Cancer Cells. Oncotarget 8 (56), 95945–95964. 10.18632/oncotarget.21606 29221178 PMC5707072

[B29] Dittmar T. Schwitalla S. Seidel J. Haverkampf S. Reith G. Meyer-Staeckling S. (2011). Characterization of Hybrid Cells Derived from Spontaneous Fusion Events between Breast Epithelial Cells Exhibiting Stem-like Characteristics and Breast Cancer Cells. Clin. Exp. Metastasis 28 (1), 75–90. 10.1007/s10585-010-9359-3 20981475

[B30] Dörnen J. Myklebost O. Dittmar T. (2020). Cell Fusion of Mesenchymal Stem/Stromal Cells and Breast Cancer Cells Leads to the Formation of Hybrid Cells Exhibiting Diverse and Individual (Stem Cell) Characteristics. Ijms 21 (24), 9636. 10.3390/ijms21249636 PMC7765946 33348862

[B31] Dörnen J. Sieler M. Weiler J. Keil S. Dittmar T. (2020). Cell Fusion-Mediated Tissue Regeneration as an Inducer of Polyploidy and Aneuploidy. Ijms 21 (5), 1811. 10.3390/ijms21051811 PMC7084716 32155721

[B32] Duelli D. Lazebnik Y. (2003). Cell Fusion: a Hidden Enemy? Cancer Cell 3 (5), 445–448. 10.1016/s1535-6108(03)00114-4 12781362

[B33] Duncan A. W. Hickey R. D. Paulk N. K. Culberson A. J. Olson S. B. Finegold M. J. (2009). Ploidy Reductions in Murine Fusion-Derived Hepatocytes. Plos Genet. 5 (2), e1000385. 10.1371/journal.pgen.1000385 19229314 PMC2636893

[B34] Dürrbaum M. Storchová Z. (2016). Effects of Aneuploidy on Gene Expression: Implications for Cancer. Febs j 283 (5), 791–802. 10.1111/febs.13591 26555863

[B35] Evans J. P. (2001). Fertilin Beta and Other ADAMs as Integrin Ligands: Insights into Cell Adhesion and Fertilization. Bioessays 23 (7), 628–639. 10.1002/bies.1088 11462216

[B36] Frade J. Nakagawa S. Cortes P. di Vicino U. Romo N. Lluis F. (2019). Controlled Ploidy Reduction of Pluripotent 4n Cells Generates 2n Cells during Mouse Embryo Development. Sci. Adv. 5 (10), eaax4199. 10.1126/sciadv.aax4199 31663024 PMC6795515

[B37] Fu Y. Li H. Hao X. (2017). The Self-Renewal Signaling Pathways Utilized by Gastric Cancer Stem Cells. Tumour Biol. 39 (4), 101042831769757. 10.1177/1010428317697577 28378630

[B38] Fu Y. Zhuang X. Xia X. Li X. Xiao K. Liu X. (2021). Correlation between Promoter Hypomethylation and Increased Expression of Syncytin-1 in Non-small Cell Lung Cancer. Ijgm 14, 957–965. 10.2147/IJGM.S294392 PMC7989540 33776474

[B39] Gamage D. G. Leikina E. Quinn M. E. Ratinov A. Chernomordik L. V. Millay D. P. (2017). Insights into the Localization and Function of Myomaker during Myoblast Fusion. J. Biol. Chem. 292 (42), 17272–17289. 10.1074/jbc.M117.811372 28860190 PMC5655506

[B40] Gauck D. Keil S. Niggemann B. Zänker K. S. Dittmar T. (2017). Hybrid Clone Cells Derived from Human Breast Epithelial Cells and Human Breast Cancer Cells Exhibit Properties of Cancer Stem/initiating Cells. BMC cancer 17 (1), 515. 10.1186/s12885-017-3509-9 28768501 PMC5541689

[B41] Gerbaud P. Pidoux G. (2015). Review: An Overview of Molecular Events Occurring in Human Trophoblast Fusion. Placenta 36 (Suppl. 1), S35–S42. 10.1016/j.placenta.2014.12.015 25564303

[B42] Getsios S. MacCalman C. D. (2003). Cadherin-11 Modulates the Terminal Differentiation and Fusion of Human Trophoblastic Cells *In Vitro* . Dev. Biol. 257 (1), 41–54. 10.1016/s0012-1606(03)00041-1 12710956

[B43] Goldenberg D. M. Zagzag D. Heselmeyer-Haddad K. M. Berroa Garcia L. Y. Ried T. Loo M. (2012). Horizontal Transmission and Retention of Malignancy, as Well as Functional Human Genes, after Spontaneous Fusion of Human Glioblastoma and Hamster Host Cells *In Vivo* . Int. J. Cancer 131 (1), 49–58. 10.1002/ijc.26327 21796629 PMC3307948

[B44] Harris H. Miller O. J. Klein G. Worst P. Tachibana T. (1969). Suppression of Malignancy by Cell Fusion. Nature 223 (5204), 363–368. 10.1038/223363a0 5387828

[B45] Hass R. von der Ohe J. Ungefroren H. (2019). Potential Role of MSC/Cancer Cell Fusion and EMT for Breast Cancer Stem Cell Formation. Cancers 11 (10), 1432. 10.3390/cancers11101432 PMC6826868 31557960

[B46] Hassan G. Seno M. (2020). Blood and Cancer: Cancer Stem Cells as Origin of Hematopoietic Cells in Solid Tumor Microenvironments. Cells 9 (5), 1293. 10.3390/cells9051293 PMC7290570 32455995

[B47] He X. Tsang T. C. Pipes B. L. Ablin R. J. Harris D. T. (2005). A Stem Cell Fusion Model of Carcinogenesis. J. Exp. Ther. Oncol. 5 (2), 101–109. 16471036

[B48] He X. Li B. Shao Y. Zhao N. Hsu Y. Zhang Z. (2015). Cell Fusion between Gastric Epithelial Cells and Mesenchymal Stem Cells Results in Epithelial-To-Mesenchymal Transition and Malignant Transformation. BMC cancer 15, 24. 10.1186/s12885-015-1027-1 25633122 PMC4318156

[B49] Hernández J. M. Podbilewicz B. (2017). The Hallmarks of Cell-Cell Fusion. Development 144 (24), 4481–4495. 10.1242/dev.155523 29254991

[B50] Huang Q. Chen H. Li J. Oliver M. Ma X. Byck D. (2014). Epigenetic and Non-epigenetic Regulation of Syncytin-1 Expression in Human Placenta and Cancer Tissues. Cell Signal. 26 (3), 648–656. 10.1016/j.cellsig.2013.11.002 24216608

[B51] Huge N. Sandbothe M. Schröder A. K. Stalke A. Eilers M. Schäffer V. (2020). Wnt Status-dependent Oncogenic Role of BCL9 and BCL9L in Hepatocellular Carcinoma. Hepatol. Int. 14 (3), 373–384. 10.1007/s12072-019-09977-w 31440992 PMC7220899

[B52] Inoue N. Ikawa M. Isotani A. Okabe M. (2005). The Immunoglobulin Superfamily Protein Izumo Is Required for Sperm to Fuse with Eggs. Nature 434 (7030), 234–238. 10.1038/nature03362 15759005

[B53] Jolly M. K. Somarelli J. A. Sheth M. Biddle A. Tripathi S. C. Armstrong A. J. (2019). Hybrid Epithelial/mesenchymal Phenotypes Promote Metastasis and Therapy Resistance across Carcinomas. Pharmacol. Ther. 194, 161–184. 10.1016/j.pharmthera.2018.09.007 30268772

[B54] Kaji K. Oda S. Shikano T. Ohnuki T. Uematsu Y. Sakagami J. (2000). The Gamete Fusion Process Is Defective in Eggs of Cd9-Deficient Mice. Nat. Genet. 24 (3), 279–282. 10.1038/73502 10700183

[B55] Katsuno Y. Derynck R. (2021). Epithelial Plasticity, Epithelial-Mesenchymal Transition, and the TGF-β Family. Dev. Cel. 56 (6), 726–746. 10.1016/j.devcel.2021.02.028 33756119

[B56] Kitao K. Tanikaga T. Miyazawa T. (2019). Identification of a post-transcriptional Regulatory Element in the Human Endogenous Retroviral Syncytin-1. J. Gen. Virol. 100 (4), 662–668. 10.1099/jgv.0.001238 30794119

[B57] Knerr I. Schubert S. W. Wich C. Amann K. Aigner T. Vogler T. (2005). Stimulation of GCMa and Syncytin via cAMP Mediated PKA Signaling in Human Trophoblastic Cells under Normoxic and Hypoxic Conditions. FEBS Lett. 579 (18), 3991–3998. 10.1016/j.febslet.2005.06.029 16004993

[B58] Koido S. (2016). Dendritic-Tumor Fusion Cell-Based Cancer Vaccines. Ijms 17 (6), 828. 10.3390/ijms17060828 PMC4926362 27240347

[B59] Krishna S. Overholtzer M. (2016). Mechanisms and Consequences of Entosis. Cell. Mol. Life Sci. 73, 2379–2386. 10.1007/s00018-016-2207-0 27048820 PMC4889469

[B60] LaBerge G. S. Duvall E. Grasmick Z. Haedicke K. Pawelek J. (2017). A Melanoma Lymph Node Metastasis with a Donor-Patient Hybrid Genome Following Bone Marrow Transplantation: A Second Case of Leucocyte-Tumor Cell Hybridization in Cancer Metastasis. PloS one 12 (2), e0168581. 10.1371/journal.pone.0168581 28146572 PMC5287451

[B61] Larsson L.-I. Bjerregaard B. Talts J. F. (2008). Cell Fusions in Mammals. Histochem. Cell Biol 129 (5), 551–561. 10.1007/s00418-008-0411-1 18351375 PMC2323033

[B62] Larsson L.-I. Bjerregaard B. Wulf-Andersen L. Talts J. F. (2007). Syncytin and Cancer Cell Fusions. The Scientific World JOURNAL 7, 1193–1197. 10.1100/tsw.2007.212 17704852 PMC5900956

[B63] Lee D. M. Chen E. H. (2019). Drosophila Myoblast Fusion: Invasion and Resistance for the Ultimate Union. Annu. Rev. Genet. 53, 67–91. 10.1146/annurev-genet-120116-024603 31283358 PMC7448821

[B64] Lee D. W. Kristiansen K. Donaldson S. H. Cadirov N. Banquy X. Israelachvili J. N. (2015). Real-time Intermembrane Force Measurements and Imaging of Lipid Domain Morphology during Hemifusion. Nat. Commun. 6, 7238. 10.1038/ncomms8238 26006266 PMC4455132

[B65] Leikina E. Gamage D. G. Prasad V. Goykhberg J. Crowe M. Diao J. (2018). Myomaker and Myomerger Work Independently to Control Distinct Steps of Membrane Remodeling during Myoblast Fusion. Dev. Cel. 46 (6), 767–780. 10.1016/j.devcel.2018.08.006 PMC6203449 30197239

[B66] Li F. Karlsson H. (2016). Expression and Regulation of Human Endogenous Retrovirus W Elements. Apmis 124 (1-2), 52–66. 10.1111/apm.12478 26818262

[B67] Li H. Feng Z. Tsang T. C. Tang T. Jia X. He X. (2014). Fusion of HepG2 Cells with Mesenchymal Stem Cells Increases Cancer-Associated and Malignant Properties: an *In Vivo* Metastasis Model. Oncol. Rep. 32 (2), 539–547. 10.3892/or.2014.3264 24926698

[B68] Li M. Li X. Zhao L. Zhou J. Cheng Y. Xu B. (2019). Spontaneous Formation of Tumorigenic Hybrids between Human Omental Adipose-Derived Stromal Cells and Endometrial Cancer Cells Increased Motility and Heterogeneity of Cancer Cells. Cell Cycle 18 (3), 320–332. 10.1080/15384101.2019.1568743 30636489 PMC6380430

[B69] Li Z. Zheng M. Zhang H. Yang X. Fan L. Fu F. (2021). Arsenic Trioxide Promotes Tumor Progression by Inducing the Formation of PGCCs and Embryonic Hemoglobin in Colon Cancer Cells. Front. Oncol. 11, 720814. 10.3389/fonc.2021.720814 34676163 PMC8523995

[B70] Liang C.-Y. Wang L.-J. Chen C.-P. Chen L.-F. Chen Y.-H. Chen H. (2010). GCM1 Regulation of the Expression of Syncytin 2 and its Cognate Receptor MFSD2A in Human Placenta1. Biol. Reprod. 83 (3), 387–395. 10.1095/biolreprod.110.083915 20484742

[B71] Lin C. Lin M. Chen H. (2005). Biochemical Characterization of the Human Placental Transcription Factor GCMa/1. Biochem. Cell Biol. 83 (2), 188–195. 10.1139/o05-026 15864327

[B72] Liu C. Xu J. Wen F. Yang F. Li X. Geng D. (2019). Upregulation of Syncytin-1 Promotes Invasion and Metastasis by Activating Epithelial-Mesenchymal Transition-Related Pathway in Endometrial Carcinoma. Ott 12, 31–40. 10.2147/ott.S191041 PMC6301305 30588028

[B73] Liu Y. Chen L. Jiang D. Luan L. Huang J. Hou Y. (2021). HER2 Promotes Epithelial-Mesenchymal Transition through Regulating Osteopontin in Gastric Cancer. Pathol. - Res. Pract. 227, 153643. 10.1016/j.prp.2021.153643 34634565

[B74] Liu Y. Tewari R. Ning J. Blagborough A. M. Garbom S. Pei J. (2008). The Conserved Plant Sterility Gene HAP2 Functions after Attachment of Fusogenic Membranes in Chlamydomonas and Plasmodium Gametes. Genes Dev. 22 (8), 1051–1068. 10.1101/gad.1656508 18367645 PMC2335326

[B75] Lokossou A. G. Toudic C. Nguyen P. T. Elisseeff X. Vargas A. Rassart É. (2020). Endogenous Retrovirus-Encoded Syncytin-2 Contributes to Exosome-Mediated Immunosuppression of T Cells. Biol. Reprod. 102 (1), 185–198. 10.1093/biolre/ioz124 31318021

[B76] Loutit J. F. Nisbet N. W. (1982). The Origin of Osteoclasts. Immunobiology 161 (3-4), 193–203. 10.1016/s0171-2985(82)80074-0 7047369

[B77] Lu X. He Y. Zhu C. Wang H. Chen S. Lin H.-Y. (2016). Twist1 Is Involved in Trophoblast Syncytialization by Regulating GCM1. Placenta 39, 45–54. 10.1016/j.placenta.2016.01.008 26992674

[B78] Lu X. Kang Y. (2009). Cell Fusion as a Hidden Force in Tumor Progression. Cancer Res. 69 (22), 8536–8539. 10.1158/0008-5472.CAN-09-2159 19887616 PMC2783941

[B79] Lu X. Kang Y. (2009). Efficient Acquisition of Dual Metastasis Organotropism to Bone and Lung through Stable Spontaneous Fusion between MDA-MB-231 Variants. Proc. Natl. Acad. Sci. 106 (23), 9385–9390. 10.1073/pnas.0900108106 19458257 PMC2695061

[B80] Lu X. Wang R. Zhu C. Wang H. Lin H.-Y. Gu Y. (2017). Fine-tuned and Cell-Cycle-Restricted Expression of Fusogenic Protein Syncytin-2 Maintains Functional Placental Syncytia. Cell Rep. 21 (5), 1150–1159. 10.1016/j.celrep.2017.10.019 29091755

[B81] Maliniemi P. Vincendeau M. Mayer J. Frank O. Hahtola S. Karenko L. (2013). Expression of Human Endogenous Retrovirus-W Including Syncytin-1 in Cutaneous T-Cell Lymphoma. PLoS One 8 (10), e76281. 10.1371/journal.pone.0076281 24098463 PMC3788054

[B82] Mangeney M. Renard M. Schlecht-Louf G. Bouallaga I. Heidmann O. Letzelter C. (2007). Placental Syncytins: Genetic Disjunction between the Fusogenic and Immunosuppressive Activity of Retroviral Envelope Proteins. Pnas 104 (51), 20534–20539. 10.1073/pnas.0707873105 18077339 PMC2154466

[B83] María G.-C. Paola I. Niki K. Mariacarmela S. Rafael B. Rosell R. (2016). Human Endogenous Retroviruses and Cancer. Cancer Biol. Med. 13 (4), 483–488. 10.20892/j.issn.2095-3941.2016.0080 28154780 PMC5250606

[B84] Matsumoto T. Wakefield L. Tarlow B. D. Grompe M. (2020). *In Vivo* Lineage Tracing of Polyploid Hepatocytes Reveals Extensive Proliferation during Liver Regeneration. Cell Stem Cell 26 (1), 34–47. 10.1016/j.stem.2019.11.014 31866222 PMC7204404

[B85] Matsuura K. Jigami T. Taniue K. Morishita Y. Adachi S. Senda T. (2011). Identification of a Link between Wnt/β-Catenin Signalling and the Cell Fusion Pathway. Nat. Commun. 2, 548. 10.1038/ncomms1551 22109522

[B86] Matteucci C. Balestrieri E. Argaw-Denboba A. Sinibaldi-Vallebona P. (2018). Human Endogenous Retroviruses Role in Cancer Cell Stemness. Semin. Cancer Biol. 53, 17–30. 10.1016/j.semcancer.2018.10.001 30317035

[B87] Medvinsky A. Smith A. (2003). Stem Cells: Fusion Brings Down Barriers. Nature 422 (6934), 823–825. 10.1038/422823a 12712184

[B88] Melzer C. von der Ohe J. Hass R. (2018). MSC Stimulate Ovarian Tumor Growth during Intercellular Communication but Reduce Tumorigenicity after Fusion with Ovarian Cancer Cells. Cell Commun Signal 16 (1), 67. 10.1186/s12964-018-0279-1 30316300 PMC6186086

[B89] Miyado K. Yamada G. Yamada S. Hasuwa H. Nakamura Y. Ryu F. (2000). Requirement of CD9 on the Egg Plasma Membrane for Fertilization. Science 287 (5451), 321–324. 10.1126/science.287.5451.321 10634791

[B90] Miyamoto H. Suzuki T. Miyauchi Y. Iwasaki R. Kobayashi T. Sato Y. (2012). Osteoclast Stimulatory Transmembrane Protein and Dendritic Cell-specific Transmembrane Protein Cooperatively Modulate Cell-Cell Fusion to Form Osteoclasts and Foreign Body Giant Cells. J. Bone Miner Res. 27 (6), 1289–1297. 10.1002/jbmr.1575 22337159

[B91] Miyamoto T. (2011). Regulators of Osteoclast Differentiation and Cell-Cell Fusion. Keio J. Med. 60 (4), 101–105. 10.2302/kjm.60.101 22200633

[B92] Mortensen K. Lichtenberg J. Thomsen P. D. Larsson L.-I. (2004). Spontaneous Fusion between Cancer Cells and Endothelial Cells. Cmls, Cell. Mol. Life Sci. 61 (16), 2125–2131. 10.1007/s00018-004-4200-2 15316661 PMC11138582

[B93] Ogle B. M. Cascalho M. Platt J. L. (2005). Biological Implications of Cell Fusion. Nat. Rev. Mol. Cell Biol 6 (7), 567–575. 10.1038/nrm1678 15957005

[B94] Orr-Weaver T. L. (2015). When Bigger Is Better: the Role of Polyploidy in Organogenesis. Trends Genet. 31 (6), 307–315. 10.1016/j.tig.2015.03.011 25921783 PMC4537166

[B95] Papahadjopoulos D. Nir S. Duzgunes N. (1990). Molecular Mechanisms of Calcium-Induced Membrane Fusion. J. Bioenerg. Biomembr 22 (2), 157–179. 10.1007/BF00762944 2139437

[B96] Pawelek J. M. Chakraborty A. K. (2008). Fusion of Tumour Cells with Bone Marrow-Derived Cells: a Unifying Explanation for Metastasis. Nat. Rev. Cancer 8 (5), 377–386. 10.1038/nrc2371 18385683

[B97] Pawelek J. M. (2005). Tumour-cell Fusion as a Source of Myeloid Traits in Cancer. Lancet Oncol. 6 (12), 988–993. 10.1016/S1470-2045(05)70466-6 16321767

[B98] Pellman D. (2007). Cell Biology: Aneuploidy and Cancer. Nature 446 (7131), 38–39. 10.1038/446038a 17330036

[B99] Petrany M. J. Millay D. P. (2019). Cell Fusion: Merging Membranes and Making Muscle. Trends Cell Biol. 29 (12), 964–973. 10.1016/j.tcb.2019.09.002 31648852 PMC7849503

[B100] Platt J. L. Cascalho M. (2019). Cell Fusion in Malignancy: A Cause or Consequence? A Provocateur or Cure? Cells 8 (6), 587. 10.3390/cells8060587 PMC6628134 31207918

[B101] Platt J. L. Cascalho M. (2019). Non-canonical B Cell Functions in Transplantation. Hum. Immunol. 80 (6), 363–377. 10.1016/j.humimm.2019.04.006 30980861 PMC6544480

[B102] Platt J. Zhou X. Lefferts A. Cascalho M. (2016). Cell Fusion in the War on Cancer: A Perspective on the Inception of Malignancy. Ijms 17 (7), 1118. 10.3390/ijms17071118 PMC4964493 27420051

[B103] Podbilewicz B. (2014). Virus and Cell Fusion Mechanisms. Annu. Rev. Cell Dev. Biol. 30, 111–139. 10.1146/annurev-cellbio-101512-122422 25000995

[B104] Poltavets V. Kochetkova M. Pitson S. M. Samuel M. S. (2018). The Role of the Extracellular Matrix and its Molecular and Cellular Regulators in Cancer Cell Plasticity. Front. Oncol. 8, 431. 10.3389/fonc.2018.00431 30356678 PMC6189298

[B105] Powell A. E. Anderson E. C. Davies P. S. Silk A. D. Pelz C. Impey S. (2011). Fusion between Intestinal Epithelial Cells and Macrophages in a Cancer Context Results in Nuclear Reprogramming. Cancer Res. 71 (4), 1497–1505. 10.1158/0008-5472.Can-10-3223 21303980 PMC3079548

[B106] Quinn M. E. Goh Q. Kurosaka M. Gamage D. G. Petrany M. J. Prasad V. (2017). Myomerger Induces Fusion of Non-fusogenic Cells and Is Required for Skeletal Muscle Development. Nat. Commun. 8, 15665. 10.1038/ncomms15665 28569755 PMC5461499

[B107] Radeczky P. Moldvay J. Fillinger J. Szeitz B. Ferencz B. Boettiger K. (2021). Bone-Specific Metastasis Pattern of Advanced-Stage Lung Adenocarcinoma According to the Localization of the Primary Tumor. Pathol. Oncol. Res. 27, 1609926. 10.3389/pore.2021.1609926 34629961 PMC8496061

[B108] Rasmussen J. P. English K. Tenlen J. R. Priess J. R. (2008). Notch Signaling and Morphogenesis of Single-Cell Tubes in the *C. elegans* Digestive Tract. Dev. Cell 14 (4), 559–569. 10.1016/j.devcel.2008.01.019 18410731 PMC2435507

[B109] Ren C. Li X. Li X. Xie Y. Fu H. Yan Z. (2018). RNAi of Grp78 May Disturb the Fusion of ICR Mouse Palate Cultured *In Vitro* . Hum. Exp. Toxicol. 37 (2), 196–204. 10.1177/0960327117692132 29233047

[B110] Saito T. Wada I. Inoue N. (2019). Sperm IZUMO1-dependent Gamete Fusion Influences Male Fertility in Mice. Ijms 20 (19), 4809. 10.3390/ijms20194809 PMC6801368 31569716

[B111] Schmidt A. Weber O. F. (2006). In Memoriam of Rudolf Virchow: a Historical Retrospective Including Aspects of Inflammation, Infection and Neoplasia. Contrib. Microbiol. 13, 1–15. 10.1159/000092961 16627956

[B112] Schumacher T. N. Schreiber R. D. (2015). Neoantigens in Cancer Immunotherapy. Science 348 (6230), 69–74. 10.1126/science.aaa4971 25838375

[B113] Searles S. C. Santosa E. K. Bui J. D. (2018). Cell-cell Fusion as a Mechanism of DNA Exchange in Cancer. Oncotarget 9 (5), 6156–6173. 10.18632/oncotarget.23715 29464062 PMC5814202

[B114] Shabo I. Svanvik J. Lindström A. Lechertier T. Trabulo S. Hulit J. (2020). Roles of Cell Fusion, Hybridization and Polyploid Cell Formation in Cancer Metastasis. Wjco 11 (3), 121–135. 10.5306/wjco.v11.i3.121 32257843 PMC7103524

[B115] Shen Y. Zhang Q. Zhang J. Lu Z. Wang A. Fei X. (2015). Advantages of a Dual-Color Fluorescence-Tracing Glioma Orthotopic Implantation Model: Detecting Tumor Location, Angiogenesis, Cellular Fusion and the Tumor Microenvironment. Exp. Ther. Med. 10 (6), 2047–2054. 10.3892/etm.2015.2821 26668594 PMC4665803

[B116] Shiokawa D. Sakai H. Ohata H. Miyazaki T. Kanda Y. Sekine S. (2020). Slow-Cycling Cancer Stem Cells Regulate Progression and Chemoresistance in Colon Cancer. Cancer Res. 80, 4451–4464. 10.1158/0008-5472.Can-20-0378 32816913

[B117] Skinner A. M. Grompe M. Kurre P. (2012). Intra-hematopoietic Cell Fusion as a Source of Somatic Variation in the Hematopoietic System. J. Cell Sci 125 (Pt 12), 2837–2843. 10.1242/jcs.100123 22393240 PMC3434805

[B118] Søe K. Andersen T. L. Hobolt-Pedersen A.-S. Bjerregaard B. Larsson L.-I. Delaissé J.-M. (2011). Involvement of Human Endogenous Retroviral Syncytin-1 in Human Osteoclast Fusion. Bone 48 (4), 837–846. 10.1016/j.bone.2010.11.011 21111077

[B119] Song K. Zhu F. Zhang H.-z. Shang Z.-j. (2012). Tumor Necrosis Factor-α Enhanced Fusions between Oral Squamous Cell Carcinoma Cells and Endothelial Cells via VCAM-1/VLA-4 Pathway. Exp. Cel. Res. 318 (14), 1707–1715. 10.1016/j.yexcr.2012.05.022 22664325

[B120] Sottile F. Aulicino F. Theka I. Cosma M. P. (2016). Mesenchymal Stem Cells Generate Distinct Functional Hybrids *In Vitro* via Cell Fusion or Entosis. Sci. Rep. 6, 36863. 10.1038/srep36863 27827439 PMC5101832

[B121] Storchova Z. Pellman D. (2004). From Polyploidy to Aneuploidy, Genome Instability and Cancer. Nat. Rev. Mol. Cell Biol 5 (1), 45–54. 10.1038/nrm1276 14708009

[B122] Strick R. Ackermann S. Langbein M. Swiatek J. Schubert S. W. Hashemolhosseini S. (2006). Proliferation and Cell-Cell Fusion of Endometrial Carcinoma Are Induced by the Human Endogenous Retroviral Syncytin-1 and Regulated by TGF-β. J. Mol. Med. 85 (1), 23–38. 10.1007/s00109-006-0104-y 17066266

[B123] Strissel P. L. Ruebner M. Thiel F. Wachter D. Ekici A. B. Wolf F. (2012). Reactivation of Codogenic Endogenous Retroviral (ERV) Envelope Genes in Human Endometrial Carcinoma and Prestages: Emergence of New Molecular Targets. Oncotarget 3 (10), 1204–1219. 10.18632/oncotarget.679 23085571 PMC3717959

[B124] Sun C. Dai X. Zhao D. Wang H. Rong X. Huang Q. (2019). Mesenchymal Stem Cells Promote Glioma Neovascularization *In Vivo* by Fusing with Cancer Stem Cells. BMC cancer 19 (1), 1240. 10.1186/s12885-019-6460-0 31864321 PMC6925905

[B125] Suzuki K. Okuno Y. Kawashima N. Muramatsu H. Okuno T. Wang X. (2016). MEF2D-BCL9 Fusion Gene Is Associated with High-Risk Acute B-Cell Precursor Lymphoblastic Leukemia in Adolescents. Jco 34 (28), 3451–3459. 10.1200/JCO.2016.66.5547 27507882

[B126] Symeonides M. Lambelé M. Roy N. Thali M. (2014). Evidence Showing that Tetraspanins Inhibit HIV-1-Induced Cell-Cell Fusion at a post-hemifusion Stage. Viruses 6 (3), 1078–1090. 10.3390/v6031078 24608085 PMC3970140

[B127] Taghizadeh S. Soheili Z.-S. Sadeghi M. Samiei S. Ranaei Pirmardan E. Kashanian A. (2021). sFLT01 Modulates Invasion and Metastasis in Prostate Cancer DU145 Cells by Inhibition of VEGF/GRP78/MMP2&9 axis. BMC Mol. Cell Biol 22 (1), 30. 10.1186/s12860-021-00367-5 34011277 PMC8135984

[B128] Toudic C. Vargas A. Xiao Y. St‐Pierre G. Bannert N. Lafond J. (2019). Galectin‐1 Interacts with the Human Endogenous Retroviral Envelope Protein Syncytin‐2 and Potentiates Trophoblast Fusion in Humans. FASEB j. 33 (11), 12873–12887. 10.1096/fj.201900107R 31499012 PMC6902695

[B129] Tug E. Yirmibes Karaoguz M. Nas T. (2020). Expression of the Syncytin-1 and Syncytin-2 Genes in the Trophoblastic Tissue of the Early Pregnancy Losses with normal and Abnormal Karyotypes. Gene 741, 144533. 10.1016/j.gene.2020.144533 32145327

[B130] Valansi C. Moi D. Leikina E. Matveev E. Graña M. Chernomordik L. V. (2017). Arabidopsis HAP2/GCS1 Is a Gamete Fusion Protein Homologous to Somatic and Viral Fusogens. J. Cel. Biol. 216 (3), 571–581. 10.1083/jcb.201610093 PMC5350521 28137780

[B131] Valcz G. Buzás E. I. Sebestyén A. Krenács T. Szállási Z. Igaz P. (2020). Extracellular Vesicle-Based Communication May Contribute to the Co-evolution of Cancer Stem Cells and Cancer-Associated Fibroblasts in Anti-cancer Therapy. Cancers 12 (8), 2324. 10.3390/cancers12082324 PMC7465064 32824649

[B132] Vargas J. N. Seemann R. Fleury J.-B. (2014). Fast Membrane Hemifusion via Dewetting between Lipid Bilayers. Soft matter 10 (46), 9293–9299. 10.1039/c4sm01577k 25330351

[B133] Wang M. Chao C.-C. Chen P.-C. Liu P.-I. Yang Y.-C. Su C.-M. (2019). Thrombospondin Enhances RANKL-dependent Osteoclastogenesis and Facilitates Lung Cancer Bone Metastasis. Biochem. Pharmacol. 166, 23–32. 10.1016/j.bcp.2019.05.005 31075265

[B134] Was H. Borkowska A. Olszewska A. Klemba A. Marciniak M. Synowiec A. (2021). Polyploidy Formation in Cancer Cells: How a Trojan Horse Is Born. Semin. Cancer Biol. S1044-579X, 00053–00055. 10.1016/j.semcancer.2021.03.003 33727077

[B135] Waza A. A. Tarfeen N. Majid S. Hassan Y. Mir R. Rather M. Y. (2021). Metastatic Breast Cancer, Organotropism and Therapeutics: A Review. Ccdt 21, 813–828. 10.2174/1568009621666210806094410 34365922

[B136] Werner B. Sottoriva A. (2018). Variation of Mutational burden in Healthy Human Tissues Suggests Non-random Strand Segregation and Allows Measuring Somatic Mutation Rates. Plos Comput. Biol. 14 (6), e1006233. 10.1371/journal.pcbi.1006233 29879111 PMC6007938

[B137] Wickner W. Schekman R. (2008). Membrane Fusion. Nat. Struct. Mol. Biol. 15 (7), 658–664. 10.1038/nsmb.1451 18618939 PMC2488960

[B138] Wu Z. Bello O. D. Thiyagarajan S. Auclair S. M. Vennekate W. Krishnakumar S. S. (2017). Dilation of Fusion Pores by Crowding of SNARE Proteins. Elife 6, e22964. 10.7554/eLife.22964 28346138 PMC5404929

[B139] Xu M.-H. Gao X. Luo D. Zhou X.-D. Xiong W. Liu G.-X. (2014). EMT and Acquisition of Stem Cell-like Properties Are Involved in Spontaneous Formation of Tumorigenic Hybrids between Lung Cancer and Bone Marrow-Derived Mesenchymal Stem Cells. PloS one 9 (2), e87893. 10.1371/journal.pone.0087893 24516569 PMC3916343

[B140] Xue J. Zhu Y. Sun Z. Ji R. Zhang X. Xu W. (2015). Tumorigenic Hybrids between Mesenchymal Stem Cells and Gastric Cancer Cells Enhanced Cancer Proliferation, Migration and Stemness. BMC cancer 15, 793. 10.1186/s12885-015-1780-1 26498753 PMC4620013

[B141] Yan H. Liu N. Zhao Z. Zhang X. Xu H. Shao B. (2012). Expression and Purification of Human TAT-P53 Fusion Protein in Pichia pastoris and its Influence on HepG2 Cell Apoptosis. Biotechnol. Lett. 34 (7), 1217–1223. 10.1007/s10529-012-0905-8 22426841

[B142] Yang Y. Zhang Y. Li W.-J. Jiang Y. Zhu Z. Hu H. (2017). Spectraplakin Induces Positive Feedback between Fusogens and the Actin Cytoskeleton to Promote Cell-Cell Fusion. Dev. Cell 41 (1), 107–120. 10.1016/j.devcel.2017.03.006 28399395

[B143] Yang Z. Yao H. Fei F. Li Y. Qu J. Li C. (2018). Generation of Erythroid Cells from Polyploid Giant Cancer Cells: Re-thinking about Tumor Blood Supply. J. Cancer Res. Clin. Oncol. 144 (4), 617–627. 10.1007/s00432-018-2598-4 29417259 PMC11813446

[B144] Yin L. Hu P. Shi X. Qian W. Zhau H. E. Pandol S. J. (2020). Cancer Cell's Neuroendocrine Feature Can Be Acquired through Cell-Cell Fusion during Cancer-Neural Stem Cell Interaction. Sci. Rep. 10 (1), 1216. 10.1038/s41598-020-58118-z 31988304 PMC6985266

[B145] Yu C. Shen K. Lin M. Chen P. Lin C. Chang G.-D. (2002). GCMa Regulates the Syncytin-Mediated Trophoblastic Fusion. J. Biol. Chem. 277 (51), 50062–50068. 10.1074/jbc.M209316200 12397062

[B146] Yu H. Liu T. Zhao Z. Chen Y. Zeng J. Liu S. (2014). Mutations in 3′-long Terminal Repeat of HERV-W Family in Chromosome 7 Upregulate Syncytin-1 Expression in Urothelial Cell Carcinoma of the Bladder through Interacting with C-Myb. Oncogene 33 (30), 3947–3958. 10.1038/onc.2013.366 24013223

[B147] Zawada K. E. Okamoto K. Kasson P. M. (2018). Influenza Hemifusion Phenotype Depends on Membrane Context: Differences in Cell-Cell and Virus-Cell Fusion. J. Mol. Biol. 430 (5), 594–601. 10.1016/j.jmb.2018.01.006 29355500 PMC5831491

[B148] Zhang D. Yang X. Yang Z. Fei F. Li S. Qu J. (2017). Daughter Cells and Erythroid Cells Budding from PGCCs and Their Clinicopathological Significances in Colorectal Cancer. J. Cancer 8 (3), 469–478. 10.7150/jca.17012 28261349 PMC5332899

[B149] Zhang L.-N. Huang Y.-H. Zhao L. (2019). Fusion of Macrophages Promotes Breast Cancer Cell Proliferation, Migration and Invasion through Activating Epithelial-Mesenchymal Transition and Wnt/β-Catenin Signaling Pathway. Arch. Biochem. Biophys. 676, 108137. 10.1016/j.abb.2019.108137 31605677

[B150] Zhang L. Ding P. Lv H. Zhang D. Liu G. Yang Z. (2014). Number of Polyploid Giant Cancer Cells and Expression of EZH2 Are Associated with VM Formation and Tumor Grade in Human Ovarian Tumor. Biomed. Res. Int. 2014, 1–9. 10.1155/2014/903542 PMC4082869 25025074

[B151] Zhang L. N. Kong C. F. Zhao D. Cong X. L. Wang S. S. Ma L. (2019). Fusion with Mesenchymal Stem Cells Differentially Affects Tumorigenic and Metastatic Abilities of Lung Cancer Cells. J. Cell Physiol 234 (4), 3570–3582. 10.1002/jcp.27011 30417342

[B152] Zhang S. Mercado-Uribe I. Liu J. (2014). Tumor Stroma and Differentiated Cancer Cells Can Be Originated Directly from Polyploid Giant Cancer Cells Induced by Paclitaxel. Int. J. Cancer 134 (3), 508–518. 10.1002/ijc.28319 23754740 PMC4175522

[B153] Zhang S. Mercado-Uribe I. Xing Z. Sun B. Kuang J. Liu J. (2014). Generation of Cancer Stem-like Cells through the Formation of Polyploid Giant Cancer Cells. Oncogene 33 (1), 116–128. 10.1038/onc.2013.96 23524583 PMC3844126

[B154] Zhang S. Xu X. Zhu S. Liu J. (2015). PGCCs Generating Erythrocytes to Form VM Structure Contributes to Tumor Blood Supply. Biomed. Research International 2015, 1–2. 10.1155/2015/402619 PMC4390101 25883960

[B155] Zhu H. Peng B. Klausen C. Yi Y. Li Y. Xiong S. (2020). NPFF Increases Fusogenic Proteins Syncytin 1 and Syncytin 2 via GCM1 in First Trimester Primary Human Cytotrophoblast Cells. FASEB j. 34 (7), 9419–9432. 10.1096/fj.201902978R 32501590

[B156] Zou B.-h. Tan Y.-h. Deng W.-d. Zheng J.-h. Yang Q. Ke M.-h. (2021). Oridonin Ameliorates Inflammation-Induced Bone Loss in Mice via Suppressing DC-STAMP Expression. Acta Pharmacol. Sin 42 (5), 744–754. 10.1038/s41401-020-0477-4 32753731 PMC8115576

